# Changes in chest CT findings of pulmonary tuberculosis after linezolid treatment

**DOI:** 10.1186/2193-1801-2-615

**Published:** 2013-11-18

**Authors:** Sanghyeon Kim, Junghoon Lee, Jongyuk Lee

**Affiliations:** Department of Radiology, Masan National Hospital, Gapo-dong, Masanhappo-gu, Changwon-si, Gyeongsangnam-do 631-710 South Korea; Department of Medicine, Masan National Hospital, Masanhappo-gu, 631-710 South Korea; Department of Radiology, Cheuk Chu Hospital, Jungang-dong, Masanhappo-gu, Changwon-si, Gyeongsangnam-do South Korea

**Keywords:** Lung, Computed tomography, Tuberculosis, Linezolid, Lung infection

## Abstract

The purpose of the present study was to evaluate the changes in successive chest CT examinations in patients with pulmonary tuberculosis who achieved culture conversion with linezolid treatment. We reviewed the follow-up CT scans of 14 patients with pulmonary tuberculosis who had sputum-culture conversion after linezolid treatment. This study evaluated cavity, centrilobular nodules, consolidation, bronchial wall thickening, calcified nodule or consolidation, bronchiectasis, irregular lines, and lung destruction. The presence of pleural thickening, pleural effusion and lymphadenopathy was recorded. Follow-up CT scans showed a partial decrease in the extent of centrilobular nodules in all cases. Consolidation was partially cleared in 8 patients and newly developed consolidation was observed in 2 patients. All of the cavities showed a decrease in size and thickness. But the cavities persisted after linezolid treatment in 8 of 9 patients. Bronchial wall thickening was completely or partially cleared in 6 patients and 5 patients, respectively. Newly developed irregular lines, lung destruction and pleural thickening were observed in 1, 1, and 3 patients, respectively. Successive chest CT examinations in patients with linezolid treatment may help in the early assessment of linezolid treatment efficacy because of its rapid availability. Early assessment of linezolid treatment efficacy will help to set up a treatment plan, such as duration of treatment or linezolid dosage. However, they may not be useful for deciding pulmonary tuberculosis activity following linezolid treatment.

## Background

Although the overall prevalence of drug-susceptible tuberculosis has reduced, the worldwide incidences of multidrug-resistant (MDR) tuberculosis and extensively drug-resistant (XDR) tuberculosis during the past decade threaten tuberculosis control since treatment is less effective, more complex, and far more costly than that for drug-susceptible disease (Frieden et al. 1993; Shah et al. 2007; Raviglione 2007). Linezolid (Zyvox, Pfizer) was approved in 2000 for drug-resistant, gram-positive bacterial infections (Leach et al. 2011). Linezolid has been shown to be effective in the treatment of MDR-tuberculosis (TB) and XDR-TB (Anger et al. 2010; Singla et al. 2012; Condos et al. 2008). However, several studies have reported side effects and toxicities, primarily bone marrow suppression and peripheral and optic neuropathy, to be limiting factors in the use of linezolid (Di Paolo et al. 2010; Lee et al. 2003). Determination of disease activity must be based on the results of sputum cultures. Although performing sputum culture is much faster nowaday, determination of drug resistance still takes several weeks. In such situation, chest CT may help in the early assessment of linezolid treatment efficacy because of its rapid availability.

The purpose of the present study was to evaluate the changes in successive chest CT examinations in patients with pulmonary tuberculosis who achieved culture conversion with linezolid treatment.

## Materials and methods

### Patients and diagnoses

Masan National Hospital approved this study. The requirement of informed consent was waived for this retrospective study.

From September 2008 through March 2013, a computer search was performed to identify all patients with pulmonary tuberculosis who were treated with linezolid. Fifty-three patients with pulmonary tuberculosis had been treated with an unchanged, failing regimen for 6 months or more before linezolid treatment and received linezolid without a change in their background treatment regimen. Among these 53 patients, we enrolled 14 patients who had sputum-culture conversion with linezolid treatment and whose serial CT scan were available. We compared two serial CT studies: the first CT scan before linezolid treatment and the second CT study after sputum-culture conversion with linezolid therapy. Conversion was defined as negative sputum samples on solid medium for 3 consecutive weeks. The time between the negative sputum and the second CT scan ranged from 28 days to 12 months (mean, 4.8 months ± 3.3). The interval between the first and second CT scans ranged from 5 to 14 months (mean, 7.2 months ± 2.6).

All 14 patients enrolled in this study were HIV-seronegative as documented by negative results of a Western blot or an enzyme-linked immunosorbent assay test.

### Image acquisition and analysis

All CT examinations were performed using a 4-detector row spiral CT scanner (Asteion; Toshiba Medical, Tokyo, Japan). None of the patients were administered an intravenous injection of contrast medium. Data were reconstructed using a bone algorithm. Scanning was performed at 120 kV with a 512 × 512 matrix. Data were reconstructed using a 2.5-mm thickness for transaxial images.

Chest CT scans were reviewed by two radiologists (SHK and JYL) with 3 and 14 years’ experience who had no knowledge of the patients’ clinical information. A final decision regarding the findings was determined by consensus. The assessed patterns of pulmonary parenchymal abnormalities included the following: centrilobular nodules (including a tree-in-bud pattern), cavity (site, number, size of the largest cavity and thickness), consolidation, bronchial wall thickening, calcified nodule or consolidation, bronchiectasis, irregular lines, and lung destruction. The descriptive terms used to interpret CT findings were as follows (Hansell et al. 2008): (i) centrilobular nodule: a nodule separated by several millimeters from the pleural surfaces, fissures, and interlobular septa; (ii) tree-in-bud pattern: centrilobular branching structures that resemble a budding tree; (iii) cavity: a gas-filled space, seen as a lucency or lowattenuation area, within pulmonary consolidation, a mass or nodule. Identification of bronchial wall thickening is largely subjective. Because bronchial wall thickening often is multifocal rather than diffuse and uniform, we compared the bronchial wall thickness of one lung region to another. The operational definition of lung destruction was one lobe or a lung with parenchymal distortion, bronchiectasis, and a decreased volume below half that of a normal lung. The cavities in areas of destroyed lung were not counted. In addition, the presence of mediastinal or hilar lymph node enlargement and pleural effusion or thickening was recorded. Enlarged lymph nodes were defined as having a short-axis diameter >1 cm on the CT scan. Each finding was classified at the second evaluation as completely or partially cleared, stable, increased, or newly developed.

## Results

### Patient demographics

Patients comprised of 10 males and 4 females (mean age, 39 years; age range, 23–59 years). Three MDR TB organism (defined as no resistance to isoniazid, rifampin, ethambutol, and pyrazinamide) and 11 XDR TB organisms were isolated. XDR TB is caused by a strain of *Mycobacterium tuberculosis* that is resistant to any type of fluoroquinolones and to at least one of the following three injectable drugs: amikacin, capreomycin or kanamycin in addition to isoniazid and rifampin and MDR TB is caused by mycobacteria resistant to at least isoniazid and rifampin (World Health Organization 2006).

### Imaging findings

#### Initial CT appearances

The most common site for the cavity includes the upper lobe in 13 patients and superior segment of the lower lobe in 9 patients. The mean number of involved lobes affected by parenchymal lesions was 4.8. In the order of their frequency, centrilobular nodules (93%), irregular lines (93%), pleural thickening (86%), bronchial wall thickening (79%), consolidation (71%), cavity (64%), bronchiectasis (64%) were observed. The average number of the cavity was 2.9, and the average size of cavities was 37.7 mm. The maximal thickness of the cavity wall was from 2 to 7 mm. The average thickness of the cavity wall was 3.8 mm. Lung destruction, calcified nodule or consolidation, lymphadenopathy and pleural effusion were observed occasionally (Table 1).

**Table 1:** **Evolution of pleural and pulmonary lesions after linezolid treatment by CT in 14 patients with pulmonary tuberculosis**

	Initial CT	Follow-up CT examination (N = 14)			
	Examination	Completely cleared	Partially cleared	Stable	New lesion
	(N = 14)				
Centrilobular nodules	13 (93)	0	13 (93)	0	0
Consolidation	10 (71)	0	8 (57)	0	2 (14)
Cavity	9 (64)	1 (7)	7 (50)	1 (7)	0
Bronchial wall thickening	11 (79)	6 (43)	5 (36)	0	0
Calcified nodule or consolidation	5 (36)	0	0	5 (36)	0
Bronchiectasis	9 (64)	0	0	9 (64)	0
Irregular lines	13 (93)	0	0	12 (86)	1 (7)
Lung destruction	6 (43)	0	0	5 (36)	1 (7)
Pleural thickening	12 (86)	0	0	9 (64)	3 (22)
Pleural effusion	1 (7)	1 (7)	0	0	0
Lymphadenopathy	2 (14)	0	0	2 (14)	0

The values in parentheses are percentages.

#### Follow-up CT appearances

**Figure 1:**
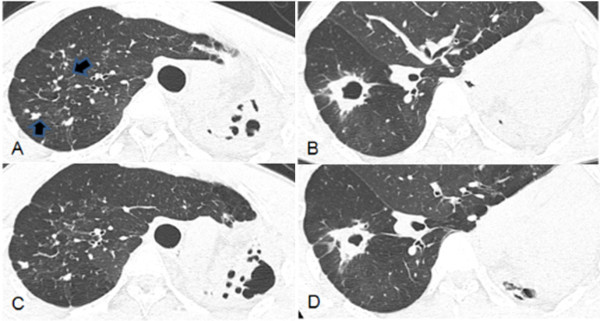
**A, B, C, D Extensively drug-resistant tuberculosis in a 33-year-old man. A** and **B**. Transaxial thin-section CT scan shows multiple centrilobular nodules (arrows) in the right upper lobe **(A)** and a cavity in the right lower lobe **(B)**. Also note destroyed left lung with mediastinal shifting. **C** and **D**. After 8 months of linezolid treatment, CT scan shows partial improvement of pulmonary nodules in the right upper lobe **(C)** and the decrease of cavity size in the right lower lobe **(D)**.

**Figure 2:**
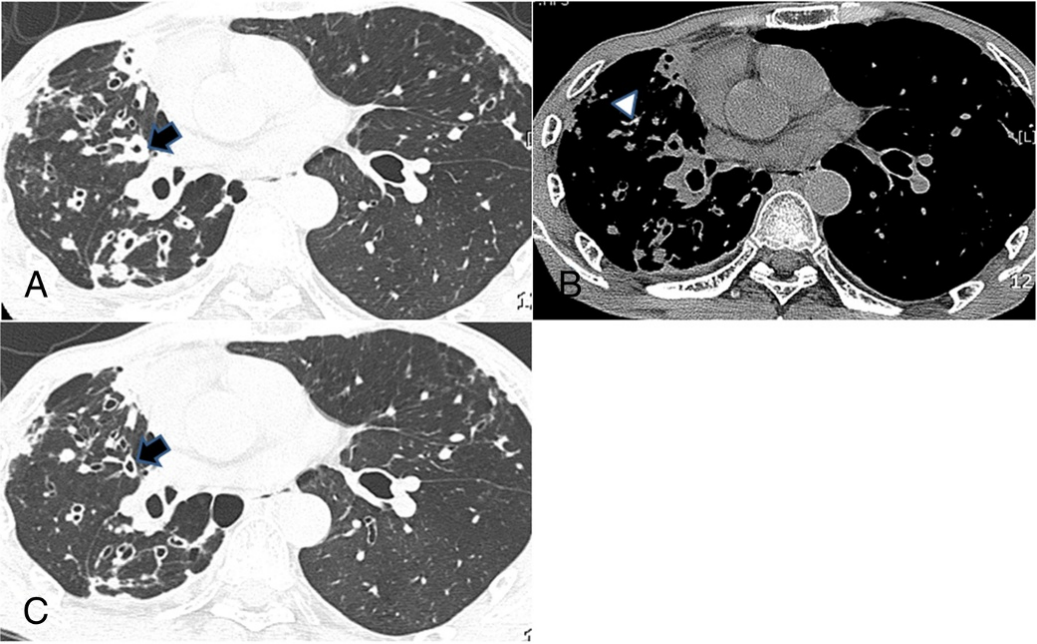
**A, B, C Extensively drug-resistant tuberculosis in a 58-year-old man.** He had been treated with antituberculosis drugs for 6 months before linezolid treatment. **A** and **B**. Transaxial thin-section CT scan shows a cavity (arrow) and bronchial wall thickening in the right upper lobe. Bronchial wall thickening and decreased volume in the right lobe are also noted. Calcified nodule (**(B)** mediastinal window, arrowhead) and pleural thickening with extrapleural fat proliferation are also observed. **C**. After 6 months of linezolid treatment, CT scan shows a persistent cavity with a decreased size and thin wall in the right upper lobe (arrow). Bronchial wall thickening improved.

Follow-up CT scans showed a partial decrease in the extent of centrilobular nodules in all cases (Figure 1). Consolidation was partially cleared in 8 patients and newly developed consolidation was observed in 2 patients. Majority of the cavities usually heal as linear or fibrotic lesions or disappear completely after conventional treatment. But, in our study, the cavities persisted after linezolid treatment in 8 of 9 patients. The average number of cavities was 1.35, and the average size of the cavity was 27 mm. The maximal thickness of the cavity wall was from 1 to 4.2 mm. The average thickness of the cavity wall was 2.1 mm. Bronchial wall thickening was completely or partially cleared in 6 patients and 5 patients, respectively (Figure 2). Newly developed irregular lines, lung destruction and pleural thickening were observed in 1, 1, and 3 patients, respectively. Pleural effusion was completely cleared and lymphadenopathy was stable in all cases.

## Discussion

Linezolid is a new antibiotic with activity against *Mycobacterium tuberculosis* and has been used to treat MDR and XDR TB with clinical improvement. Linezolid’s safety and tolerability are limited by the dose- and duration-dependent occurrence of reversible myelosuppression and peripheral and optic neuropathy (Bressler et al. 2004; Rucker et al. 2006). Early assessment of linezolid treatment efficacy is necessary. In addition to the diagnosis of TB, CT is useful in determining disease activity (Jeong & Lee 2008). In the series by Lee et al. (Lee et al. 1996), 80% of patients with active disease and 89% of those with inactive disease were correctly differentiated on high-resolution CT.

CT is more sensitive than chest radiography in the detection and characterization of both subtle localized or disseminated parenchymal disease (McGuinness et al. 1992; Kim et al. 1997). The most common CT findings of active pulmonary TB are centrilobular small nodules, branching linear and nodular opacities (tree-in-bud sign), patchy or lobular areas of consolidation, and cavitation (Lee et al. 1993). Lesions including areas of irregular lines, calcified nodules along with distortion of bronchovascular bundles, bronchiectasis, pericicatricial emphysema, or a combination of the above were presumed to be inactive (Lee & Im 1995). Lee et al. (Lee et al. 1996) showed that calcified nodule or consolidation, irregular lines, parenchymal bands, and pericicatricial emphysema were unique findings that were observed only in patients with inactive pulmonary tuberculosis. However, in our study, the initial CT study showed these findings in most of the patients. These findings can be explained based on the patients’ the clinical history, namely, a chronic course of the disease. All of the patients had been treated with antituberculosis drugs for 6 months or more before enrollment in our study. Radiological findings might show inadvertently progressive features with an ongoing chronic TB infection.

The most important finding for making an accurate diagnosis of active tuberculosis on CT is the endobronchial spread of infection. The centrilobular small nodules and tree-in-bud sign reflect the presence of endobronchial spread (Leung 1999). With more extensive disease, coalescence of the centrilobular opacities occurs, resulting in focal areas of bronchopneumonia. In our study, these findings of infection, such as centrilobular nodules, consolidation and bronchial wall thickening were commonly observed in the initial CT scan. Bronchial wall thickening disappeared completely in 6 patients or partially in 5 patients. However, centrilobular nodules and consolidation were not completely cleared, and they persisted in all patients except for two patients in spite of negative sputum following linezolid treatment. Two patients showed newly developed consolidation but one of the two patients was available for further follow-up CT that did not show a significant change.

Cavities are the radiologic hallmark of reactivation TB (Miller & Miller 1993). Majority of the cavities usually heal as linear or fibrotic lesions or disappear completely. However, complete anatomical clearing of the cavities after adequate therapy dose not always take place. Some cavities persist even after antituberculous therapy. In 8 of 9 patients with cavities, the cavities became smooth and smaller with linezolid treatment but they persisted. Thus, CT findings of pulmonary TB such as persistent centrilobular nodules, consolidation or cavities may cause confusion regarding pulmonary tuberculosis activity after linezolid treatment.

There are some reports about the difference in CT findings between drug-sensitive TB and XDR/MDR TB. Multiple cavities and findings of chronicity such as bronchiectasis and calcified granulomas are more common in patients with MDR TB than drug-sensitive TB (Kim et al. 2004; Chung et al. 2006). CT findings of pulmonary XDR TB are similar to those of non XDR MDR-TB (Lee et al. 2010; Cha et al. 2009). Similarly, there is no significant difference in imaging findings between patients with XDR TB and MDR TB in our study.

This study has some limitations. First, there might be some selection bias in this study, because not all of the patients with pulmonary tuberculosis underwent a CT scan. Second, the population studied was small, and hence, there were no controls.

In conclusion, successive chest CT examinations in patients with linezolid treatment may help in the early assessment of linezolid treatment efficacy because of its rapid availability. Early assessment of linezolid treatment efficacy will help to set up a treatment plan, such as duration of treatment or linezolid dosage. However, they may not be useful for deciding pulmonary tuberculosis activity following linezolid treatment.
